# MRC1 and LYVE1 expressing macrophages in vascular beds of GNAQ p.R183Q driven capillary malformations in Sturge Weber syndrome

**DOI:** 10.1186/s40478-024-01757-4

**Published:** 2024-03-26

**Authors:** Sana Nasim, Colette Bichsel, Stephen Dayneka, Robert Mannix, Annegret Holm, Mathew Vivero, Sanda Alexandrescu, Anna Pinto, Arin K. Greene, Donald E. Ingber, Joyce Bischoff

**Affiliations:** 1 Vascular Biology Program, Boston Children’s Hospital and Harvard Medical School, Boston, MA 02115 USA; 2 Department of Surgery, Boston Children’s Hospital and Harvard Medical School, Boston, MA 02115 USA; 3 CSEM SA, Hegenheimermattweg 167 A, 4123 Allschwil, Switzerland; 4 Department of Plastic & Oral Surgery, Boston Children’s Hospital and Harvard Medical School, Boston, MA 02115 USA; 5 Department of Pathology, Boston Children’s Hospital and Harvard Medical School, Boston, MA 02115 USA; 6 Department of Neurology, Boston Children’s Hospital and Harvard Medical School, Boston, MA 02115 USA; 7 Wyss Institute for Biologically Inspired Engineering at Harvard University, Boston, MA 02215 USA; 8 Harvard John A. Paulson School of Engineering and Applied Sciences, Harvard University, Cambridge, MA 02139 USA

**Keywords:** Capillary malformation, *GNAQ*, Sturge Weber syndrome, Vascular malformation, Macrophages, Leukocyte adhesion, ICAM1

## Abstract

Sturge-Weber syndrome (SWS), a neurocutaneous disorder, is characterized by capillary malformations (CM) in the skin, brain, and eyes. Patients may suffer from seizures, strokes, and glaucoma, and only symptomatic treatment is available. CM are comprised of enlarged vessels with endothelial cells (ECs) and disorganized mural cells. Our recent finding indicated that the R183Q mutation in ECs leads to heightened signaling through phospholipase Cβ3 and protein kinase C, leading to increased angiopoietin-2 (ANGPT2). Furthermore, knockdown of ANGPT2, a crucial mediator of pro-angiogenic signaling, inflammation, and vascular remodeling, in EC-R183Q rescued the enlarged vessel phenotype in vivo. This prompted us to look closer at the microenvironment in CM-affected vascular beds. We analyzed multiple brain histological sections from patients with *GNAQ*-R183Q CM and found enlarged vessels devoid of mural cells along with increased macrophage-like cells co-expressing MRC1 (CD206, a mannose receptor), CD163 (a scavenger receptor and marker of the monocyte/macrophage lineage), CD68 (a pan macrophage marker), and LYVE1 (a lymphatic marker expressed by some macrophages). These macrophages were not found in non-SWS control brain sections. To investigate the mechanism of increased macrophages in the perivascular environment, we examined THP1 (monocytic/macrophage cell line) cell adhesion to EC-R183Q versus EC-WT under static and laminar flow conditions. First, we observed increased THP1 cell adhesion to EC-R183Q compared to EC-WT under static conditions. Next, using live cell imaging, we found THP1 cell adhesion to EC-R183Q was dramatically increased under laminar flow conditions and could be inhibited by anti-ICAM1. ICAM1, an endothelial cell adhesion molecule required for leukocyte adhesion, was strongly expressed in the endothelium in SWS brain histological sections, suggesting a mechanism for recruitment of macrophages. In conclusion, our findings demonstrate that macrophages are an important component of the perivascular environment in CM suggesting they may contribute to the CM formation and SWS disease progression.

**Supplementary Information:**

The online version contains supplementary material available at 10.1186/s40478-024-01757-4.

## Introduction

Capillary malformations (CM) are characterized by abnormally dense and tortuous capillary-venule-sized blood vessels. CM are categorized as slow flow vascular malformations, and some can cause overgrowth of underlying soft tissue. Non-syndromic, cutaneous CM are often located on the face and neck and are known as port wine stains or port wine birthmarks. In Sturge-Weber syndrome (SWS), CM are found in the leptomeninges of the brain, the choroid of the eye, and skin, in a distinctive facial pattern [21]. The CM in the brain leptomeninges can lead to debilitating neurological symptoms such as seizures, hemiparesis, and migraines [31, 32]. In SWS, perfusion imaging also shows impaired venous drainage in the affected brain regions resulting in impaired, dilated arterial perfusion [31]. The risk of neurological involvement is correlated with the location and extent of the port wine stains. SWS risk is ~ 1/4 if the facial CM presents in a hemifacial pattern including the forehead and upper eyelid.

A somatic missense mutation in the *GNAQ* gene, which encodes the G-protein subunit α_q_, consists of arginine (R) at the amino acid position of 183 replaced by the glutamine (Q). The p.R183Q mutation is found in 90% of syndromic and non-syndromic CM [9, 20, 22]. Point mutations in the closely related *GNA11* gene have been found in three cases of CM on the extremities and these cases were not reported to have SWS [8]. Another report showed one patient negative for *GNAQ* mutation but with a somatic mutation in *GNB2* [11]. The *GNAQ* p.R183Q mutation is postulated to cause constitutive activation of Gα_q_ and in turn activation of phospholipase Cβ3. One of the consequences of this overactivation is an increased production of angiopoietin-2 (ANGPT2), a key mediator of pro-angiogenic signaling and inflammation [12, 14]. Moreover, high levels of endothelial HIF-1α and HIF-2α in CM-affected leptomeninges portend the presence of sustained angiogenic signals [6].

Our rationale for this study was to determine if and how the perivascular microenvironment surrounding CM vessels might be affected by the *GNAQ* R183Q mutation shown to be present in endothelial cells (ECs) in skin and brain CMs [9, 15]. We hypothesize the overly active Gα_q_ leads to abnormal endothelial signaling and this in turn exerts paracrine and/or juxtracrine effects on nearby cells including mural cells, parenchymal cells, and other locally present cells (tissue-specific or recruited). Further, we speculate that these interactions play an important role in CM and SWS pathogenesis.

To address this, we analyzed multiple SWS and non-SWS brain specimens and found enlarged vessels as expected, an increased number of vessels lacking mural cells, and increased levels of macrophages expressing MRC1, CD163, CD68, and LYVE1 in SWS brain sections. Using in vitro assays, mutant ECs promote macrophage adhesion, and this adhesion was reduced by anti-human leukocyte adhesion molecule-1 (ICAM1) antibody. These observations add another level of complexity to CMs, and the presence of the MRC1^+^/CD68^+^/LYVE1^+^ cells should be further studied in animal models.

## Materials and methods

### H&E and Martius Scarlet Blue staining

Human SWS brain and non-SWS brain specimens (Table 1) were obtained under a human subject protocol (IRB-P00003505) approved by the Committee on Clinical Investigation at the Boston Children’s Hospital. Informed consent was obtained from each patient according to the IRB protocol. In brief, 5 µm formalin fixed paraffin embedded (FFPE) tissue sections were deparaffinized and stained with hematoxylin and eosin (H&E). Sectioning and H&E staining were performed by the Boston Children’s Hospital Department of Pathology. Tissue sections from the same human specimens were sent for Martius Scarlet Blue staining to iHisto (Salem, MA). MoticEasyScan Infinity 60 microscope was used for bright field scanning of each tissue section slide.

**Table 1:** Patient demographic information for brain specimens

	Age	Sex	Diagnosis	Mutation	Mutant Allelic frequency (%)	Location of the specimen
*Patients with SWS*						
1	2 years	Female	Sturge-Weber syndrome	*GNAQ* R183Q	2.96	Lateral temporal lobe
2	4 years	Female	Sturge-Weber syndrome	*GNAQ* R183Q	4.59	Left lateral temporal lobe
3	14 years	Male	Sturge-Weber syndrome	*GNAQ* R183Q	0.15	Right temporal lobe
4	4 years	Male	Sturge-Weber syndrome	*GNAQ* R183Q	2.5	Right posterior lateral temporal tip
*Patients without SWS*						
1	3 months	Female	N.A	N.A	–	Right medial temporal lobe
2	3 years	Male	N.A	N.A	–	Left frontal lobe
*Patient with inflamed brain*						
1	9 days	Female	Multiple cardiac anomalies	N.A	–	Right frontal lobe

### Immunofluorescence staining

Immunostaining was performed on FFPE sections, which were deparaffinized and incubated in citrate buffer at pH 6.0 for 20 min at 95 °C for antigen retrieval. Tissue sections were then blocked in Tris-NaCl buffer with 0.5% blocking reagent (Cat# FP1012, Perkin Elmer) for 30 min before incubating with primary antibodies (Additional file 7: Table S1) for one hour at room temperature or overnight at 4 °C. After washing in Tris-NaCl buffer with 0.05% Tween-20 (BioRad), the sections were incubated with secondary antibodies (Additional file 7: Table S1) for one hour at room temperature, washed, counterstained with 4′,6-diamidino-2-phenylindole (DAPI) (Cat# R37606, Invitrogen) and mounted. Images were taken on the Zeiss laser scanning 880 confocal microscope and analyzed in either ZenBlack or Fiji.

### Image quantification

Vessel characteristics such as circumference and area were assessed by hand using the outline tool in Fiji. For vessel area and circumference quantification, six to seven images from each specimen were measured. For mural cell coverage quantification, vessel circumference was measured from ten to twelve images from each specimen.

For automatic colocalization, the Pearson Correlation Coefficient (PCC) and the Manders Overlap Coefficients served as the primary indicators for fluorescent colocalization. The PCC serves to represent how closely two markers follow a simple linear relationship. The Manders Overlap Coefficients–denoted by tM1 and tM2–reveal the proportion of cell area that shows colocalization of markers in comparison to the total measured fluorescence of one marker [18]. Next, the Costes Method of colocalization analysis was applied using the Coloc2 plugin in Fiji [7]. For each case, the colocalization analyses were applied to each of the six to seven images captured from each sample. In the case of triple colocalization, analyses were performed by applying the automatic thresholding described above in a stepwise manner. First, the colocalization of two markers was determined, resulting in a new image that colorized individual markers in addition to the areas of colocalization. The colocalized areas were then isolated and in a final step, the automatic analysis was performed between the third marker and the colocalized areas.

### Droplet digital polymerase chain reaction

Droplet digital PCR (ddPCR) was used to measure the mutant allelic frequency in tissue sections as previously described [2]. Briefly, DNA was isolated from FFPE tissue sections using a FormaPure kit (Cat# C16675, Beckman Coulter). ddPCR with probes (Additional file 8: Table S2) to detect the *GNAQ* R183Q mutation was run with 15 ng DNA per sample along with ddPCR SuperMix (Cat# 1,863,010, BioRad). The droplet fluorescence was read in a QX200 droplet reader and analyzed in QuantaSoft software (BioRad).

### Cell culture

A *GNAQ* R183Q cellular model was generated by CRISPR editing of one *GNAQ* allele in telomerase immortalized human aortic ECs (Vivero et al. manuscript submitted). Cells were cultured on 0.1 µg/cm^2^ fibronectin (Cat#FC010-10MG, Millipore Sigma-Aldrich) coated plates at 15,000 to 20,000 cell/cm^2^ in endothelial cell growth medium-2 (EGM2, Lonza), which contains endothelial cell growth medium-2 (EBM2), SingleQuot supplements (all except hydrocortisone and GA-1000), 10% heat-inactivated fetal bovine serum (FBS, Cytiva), and 1X glutamine-penicillin–streptomycin (Thermo Fisher). THP1 cells were commercially purchased from ATCC (Cat#TIB-202). U937, a second monocytic cell line, were generously shared by Dr. Dipak Panigrahy. Cells were grown in suspension on untreated plates at 30,000 to 50,000 cell/cm^2^ density in DMEM/F-12 50/50 1X media (Cat#10-092-CV) supplemented with 10% FBS.

### THP1 and U937 cell adhesion assay

Leukocyte endothelial adhesion assays (Cat# CBA210, Cell Biolabs) were performed in accordance with the manufacturer's protocol. Briefly, 50,000 ECs (EC-WT or EC-R183Q) were plated in 48 well plates and cultured overnight in complete EGM2 media. 50,000 THP1 or U937 cells were pretreated with Leuko Tracker Solution in EBM2 before use in assay. ECs were incubated with labeled THP1 or U937 cells for 90 min before fluorescence reading at 480/520 nm on a Synergy H1 microplate reader (BioTek).

### Live imaging of THP1 cell adhesion

The recruitment of monocytes was investigated on the basis of previous reports [23, 26]. Briefly, µSlide I^0.4^ Luer ibiTreat (Cat# 80,176, Ibidi) were coated with 1 µg/cm^2^ fibronectin in 0.1 M bicarbonate buffer (pH 9.4). 2.5 million cells per milliliter (either EC-WT or EC-R183Q) were seeded in complete EGM2 media and were allowed to adhere overnight. On the day of the experiment, THP1 cells were stained with CellTracker™ Red CMTPX dye (Cat# 34,552, Thermo Fisher Scientific) for 20 min at 37 °C and washed in serum-free EBM2 media. 300,000 labeled THP1 cells per milliliter were used for all experiments. For the anti-ICAM1 specific experiments, EC-R183Q were seeded and treated with 10 µg/ml human anti-ICAM1 antibody (Cat# BE0020-2, BioXCell) or IgG2A (Cat#BE0085, BioXCell) isotype control 30 min prior to exposing the treated monolayer of EC-R183Q to labeled THP1 cells. A flow rate of 0.5 ml/min, corresponding to ~ 0.8 dyne/cm^2^ was setup using a tabletop syringe pump with a BD 20 ml syringe Luer-Lock tip. After 5 min of recording, a switch system was used to deliver pre-stained THP1 cells under continuous uninterrupted flow for 30 min. An image was generated every 5 s for 30 min, using combinations of phase contrast and fluorescence imaging with a Nikon TiEclipse Inverted Microscope using 10X objective. For quantification, the TrackMate plugin in Fiji was used. Within the TrackMate plugin, the StarDisk detector was used to integrate a state-of-the-art segmentation algorithm, and Simple Linear Assignment Problem tracker to track the moving vs. adherent THP1 cells [10]. At the end of each analysis, quantification of spot per frame was used to present THP1 cell adhesion as mean adherent monocytes per mm^2^ ± standard error mean (SEM) for 10, 20, and 30 min. 6 independent experiments were run for each EC-WT and EC-R183Q.

### Cytokine array

EC-WT and EC-R183Q were seeded 20,000 cells/cm^2^ and cultured for 48 h. Cells were switched to 2% FBS in EBM2 for 24 h. Conditioned media was collected and stored at − 80 °C. The cytokine array was performed on conditioned media using Proteome Profiler human cytokine array (Cat# ARY005B, R&D Systems) in accordance with the manufacturer protocol. Signals were detected by enhanced chemiluminescence and densitometric analysis was conducted using Fiji. 3 independent experiments were run for each EC-WT and EC-R183Q.

### Statistical analysis

One-way ANOVA was performed followed by a post-hoc Kruskal–Wallis or Dunn multiple comparison test. *T*-tests were performed when one comparison was made, e.g. EC-WT versus EC-R183Q. Shapiro–Wilk test for normality was applied. Significance was considered as *p* < 0.05. Data is reported as mean ± SEM. All tests were run with the GraphPad Prism 9.2 software.

## Results

### CM blood vessels are enlarged and some lack mural cell coverage

**Fig. 1:**
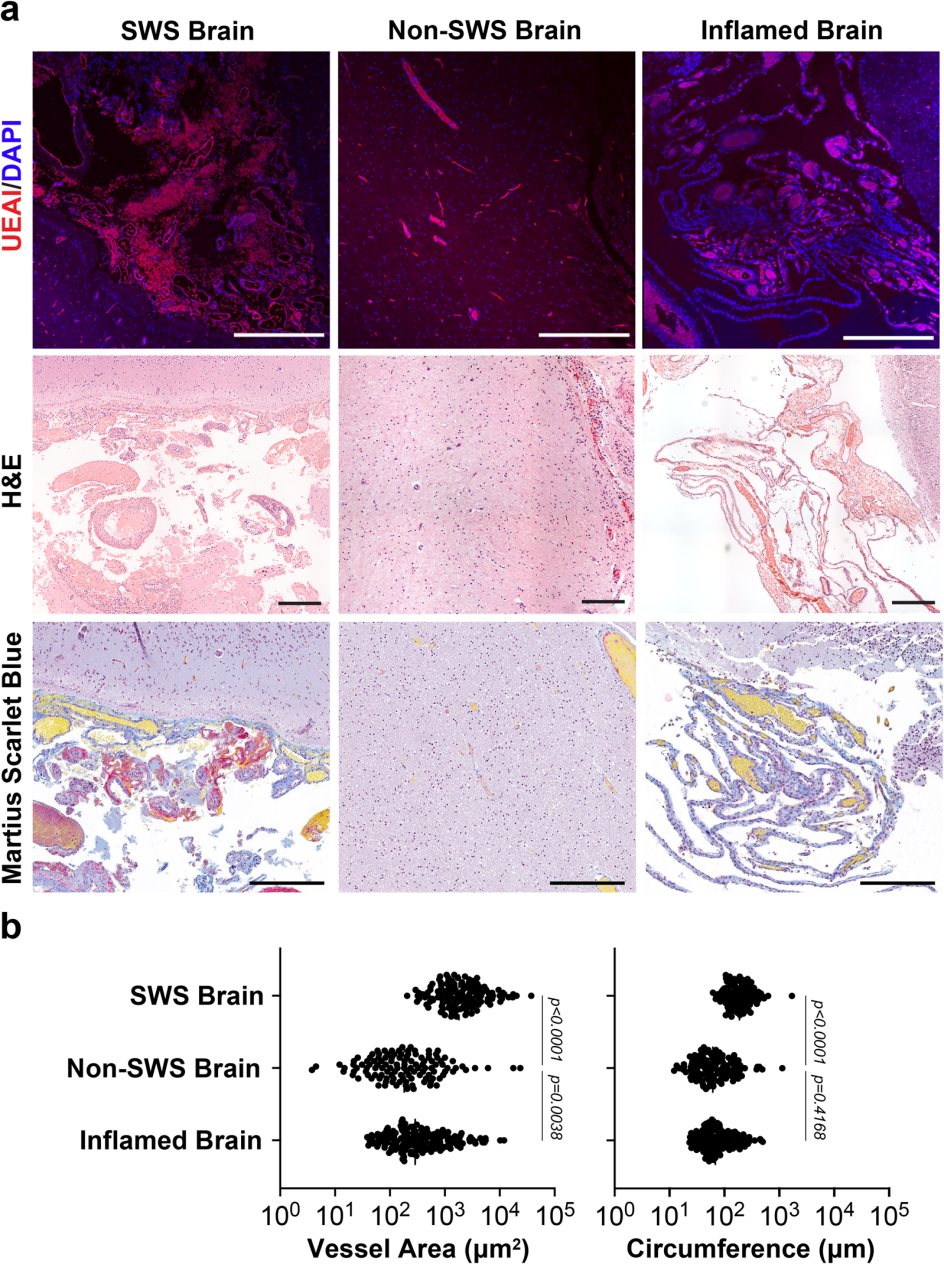
Characterization of blood vessels in the Sturge-Weber brain. **a** Endothelial staining with Ulex europaeus agglutinin-I (UEAI, red) and nuclei counterstaining with DAPI (blue) (top), scale bar = 500 µm; Hematoxylin and Eosin (H&E) (middle), scale bar = 50 mm; and Martius Scarlet Blue (bottom) staining, scale bar = 500 µm, fibrin (red), collagen (blue), erythrocytes (yellow), and nuclei (blue/black) in SWS brain, non-SWS brain, and inflamed brain. **b** Quantification of the vessel area (µm^2^) and circumference (µm) in the SWS brain (n = 4), non-SWS brain (n = 2) and inflamed brain (n = 1) specimens. The *p*-values were calculated by One-way ANOVA followed by Dunn’s multiple comparison test

CMs consist of clusters of abnormal blood vessels. To characterize these abnormalities, we examined tissue sections from four SWS brains and compared them to non-SWS brain tissue sections (Table 1). The CM regions had overabundant blood vessels in disarray as compared to unaffected tissue stained for EC specific marker, Ulex europaeus agglutinin-I (UEAI) (Fig. 1a). Vessels were enlarged, as previously reported for skin CM [17, 25], shown by measuring vessel area and circumference (Fig. 1b). Many of the vessels in SWS brain showed accumulation of fibrin, shown by Martius Scarlet Blue staining, surrounding the enlarged blood vessels compared to non-SWS brain (Fig. 1a). This suggests permeability or leakiness in the CM endothelium.

**Fig. 2:**
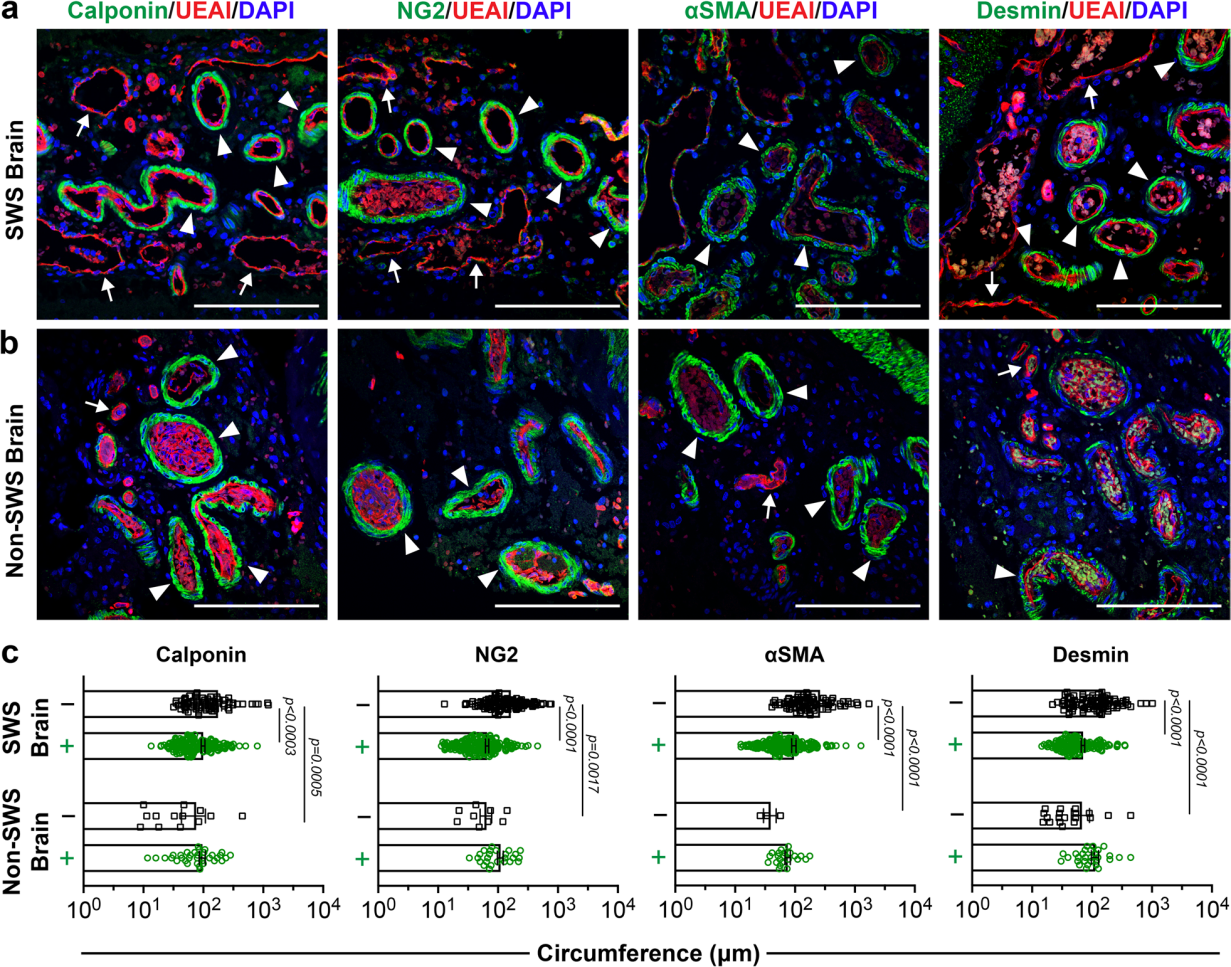
Characterization of mural cells in brain specimens. Antibody staining for calponin, neuron-glia antigen 2 (NG2), alpha smooth muscle actin (αSMA), desmin (green), UEAI (red), and nuclei staining with DAPI (blue) in **a** SWS brain and **b** non-SWS brain. Scale bar = 50 µm. White arrows point at vessels without mural cells, whereas arrowheads point to vessels with mural cell coverage. **c** Quantification of vessel circumference (µm) in SWS brain (n = 4) and non-SWS brain (n = 2). ‘+’ (green) indicates the vessels surrounded by mural cells, and ‘−’ (black) indicates the vessels that lack mural cell coverage. The p-values were calculated by a two-tailed, non-parametric Mann–Whitney test

Next, we examined the mural cell coverage by immunostaining with antibodies for mural cell markers: calponin, neuron-glia antigen 2 (NG2), alpha-smooth muscle actin (αSMA), and desmin in SWS brain (Fig. 2a) and non-SWS brain sections (Fig. 2b). These are commonly used markers to identify mural cells such as pericytes and smooth muscle cells. We found vessels that lacked mural cells were significantly enlarged compared to those encased in mural cells; this result held with all 4 markers. Non-SWS brain vessels showed no difference in size between mural cell covered and non-covered vessels (Fig. 2c). Moreover, blood vessels lacking mural cell coverage in non-SWS brain are significantly smaller in circumference compared to the mural cell negative blood vessels in SWS brain (Fig. 2c). These findings link the enlarged vessel phenotype to mural cell assembly around blood vessels in CM.

### *CM-affected vascular beds in SWS brain contain phagocytic macrophages*:

**Fig. 3:**
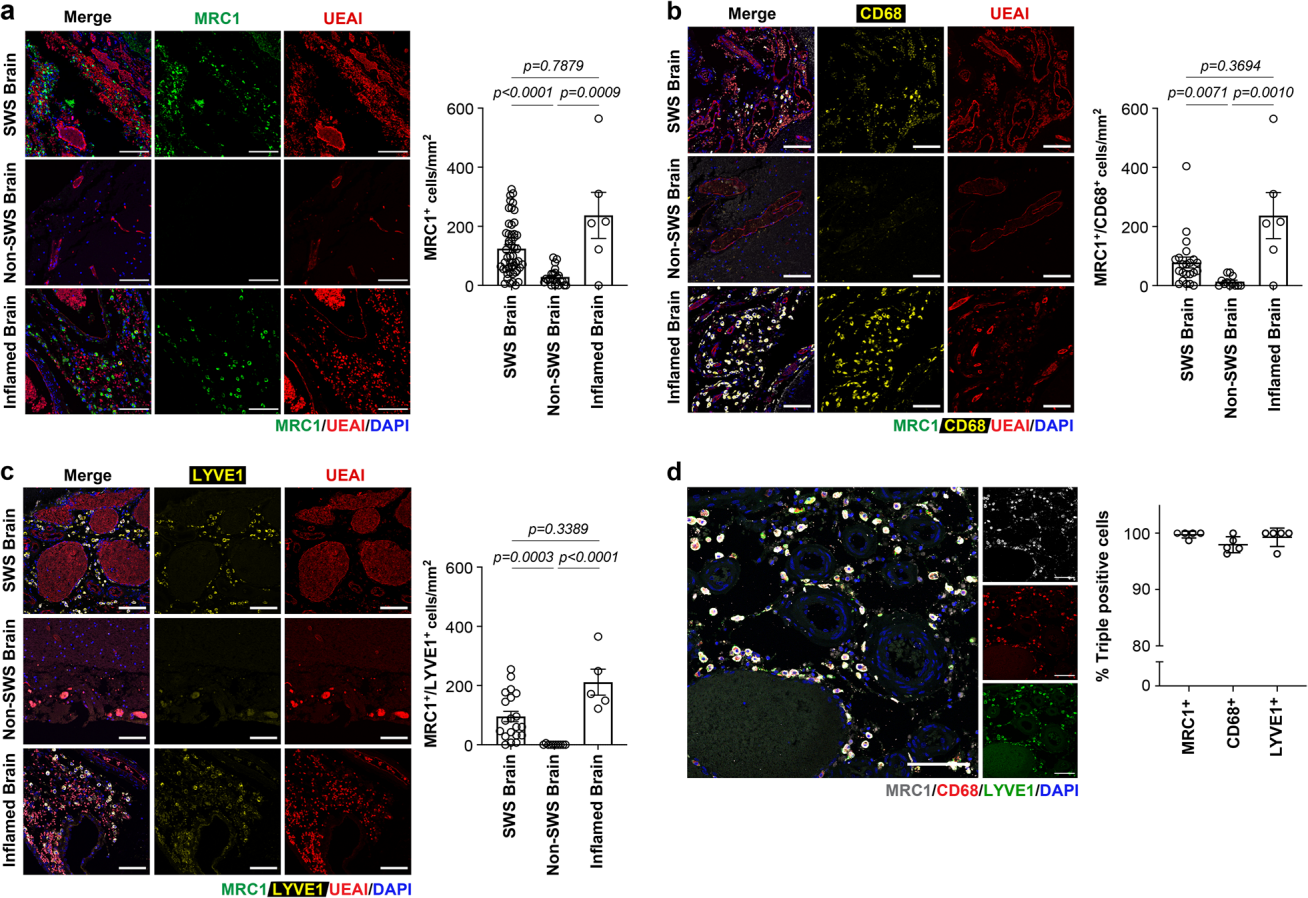
Macrophages in the perivascular space of Sturge-Weber brain. **a** Staining for MRC1 (green), UEAI (endothelium, red) and counterstaining for DAPI (blue) in SWS brain sections (top), non-SWS brain sections (middle), and inflamed brain sections (bottom). Quantification of MRC1^+^ cells (right). **b** Co-staining for CD68 (yellow), MRC1 (grey), UEAI (red) and counterstaining for DAPI (blue) in the SWS brain (top), non-SWS brain (middle), and inflamed brain (bottom). Quantification of co-labeled MRC1^+^ and CD68^+^ cells (right) **c** Co-staining of lymphatic vessel endothelial hyaluronan receptor 1 (LYVE1, yellow), MRC1 (grey), UEAI (red) and counterstaining for DAPI (blue) in the SWS brain (top), non-SWS brain (middle), and inflamed brain (bottom). Quantification of co-labeled MRC1^+^ and LYVE1^+^ cells (right). The *p*-values were calculated by One-way ANOVA test with Dunn’s multiple comparison test. **d** Triple staining of MRC1^+^, CD68^+^, and LYVE1^+^ in the SWS brain. Quantification (right) shows more than 95% of cells are triple positive for MRC1, CD68, and LYVE1. See Table 1 for information on brain specimens. SWS brain (n = 4), non-SWS brain (n = 2) and inflamed brain (n = 1) specimens. All scale bars are 50 µm

We next investigated whether macrophages are present in CM-affected SWS vascular beds. Strikingly, we observed a high number of cells positive for Mannose Receptor 1 (MRC1/CD206), a macrophage marker, in the perivascular areas in CM-affected SWS brain specimens (Fig. 3a). MRC1 acts as a phagocytic receptor by binding high mannose structures on pathogens and it is associated with the M2 macrophage phenotype. The density of MRC1^+^ cells was variable within and between specimens (Additional file 1: Fig. S1), but overall, there was a significant increase of MRC1^+^ cells in CM-affected SWS brain compared to non-SWS brain specimens. To put the MRC1^+^ numbers into perspective, we also compared them to a brain specimen with ongoing inflammation. Compared to this positive control, MRC1^+^ cells were similarly abundant in CM-affected SWS vascular beds. Moreover, the MRC1^+^ cells were also CD163^+^, which is expressed by monocytes and macrophages and found on tumor associated macrophages as well as M2 macrophages (Additional file 2: Fig. S2).

**Fig. 4:**
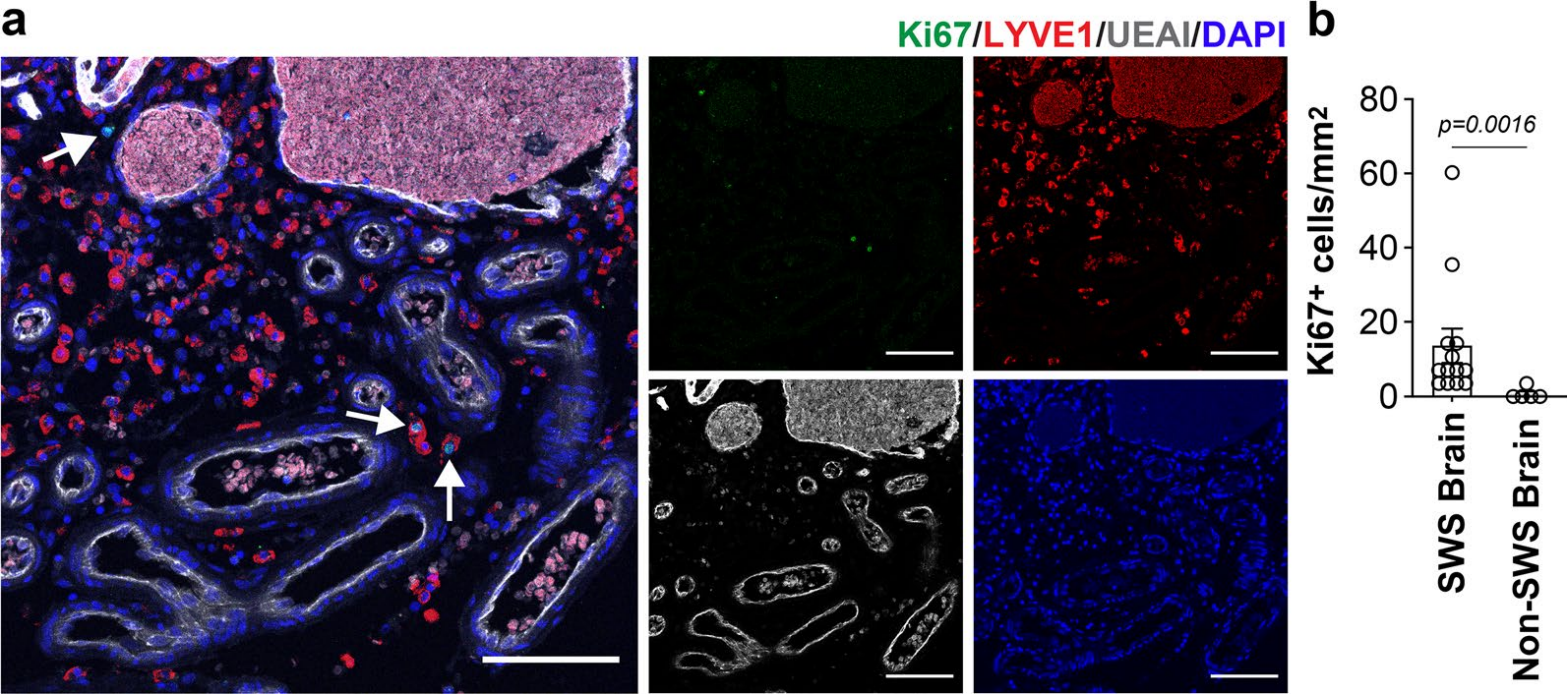
Some LYVE1^+^ cells in Sturge Weber brain are Ki67^+^. **a** Ki67 (green), LYVE1 (red), UEAI (grey) and counterstaining for DAPI (blue). The white arrows point to Ki67^+^ and LYVE1^+^ cells. Separate channels for each marker: Ki67 (top left), LYVE1 (top right), UEAI (bottom left) and DAPI (bottom right). Scale bar = 50 µm. **b** Quantification of Ki67^+^ cells in SWS brain (n = 4) and non-SWS brain (n = 2) specimens. *P*-value was calculated by two-tailed *t*-test

High-magnification views showed MRC1^+^ cells phagocytosing red blood cells in the perivascular space (Additional file 3: Fig. S3), which indicates macrophage function. Co-staining with anti-αSMA confirmed that MRC1^+^ cells are distinct from perivascular αSMA^+^ cells (Additional file 4: Fig. S4). To further characterize the MRC1^+^ cells, we co-stained with the pan-macrophage marker, Cluster of Differentiation 68 (CD68), and saw a significant increase in cells expressing both MRC1 and CD68 compared to non-SWS brain (Fig. 3b). Lymphatic vessel endothelial hyaluronan receptor-1 (LYVE1), a lymphatic marker that is also expressed in alternatively polarized macrophages, was also significantly increased and co-localized with MRC1^+^ cells in SWS brain compared to non-SWS brain (Fig. 3c). We further examined colocalization of all three markers—MRC1, CD68, and LYVE1, and found ~ 95% of the cells to be triple positive for all three markers (Fig. 3d). Next, we immunostained for the proliferating cell marker Ki67 to assess whether the macrophages might be dividing. Some LYVE1^+^ cells were Ki67^+^ in the SWS brain, while almost none were detected in non-SWS brain sections (Fig. 4). This suggests that some of the LYVE1^+^ cells, which we show are MRC1^+^/CD68^+^, are proliferating in the perivascular space.

### Neutrophils are sparse in the CM microenvironment

**Fig. 5:**
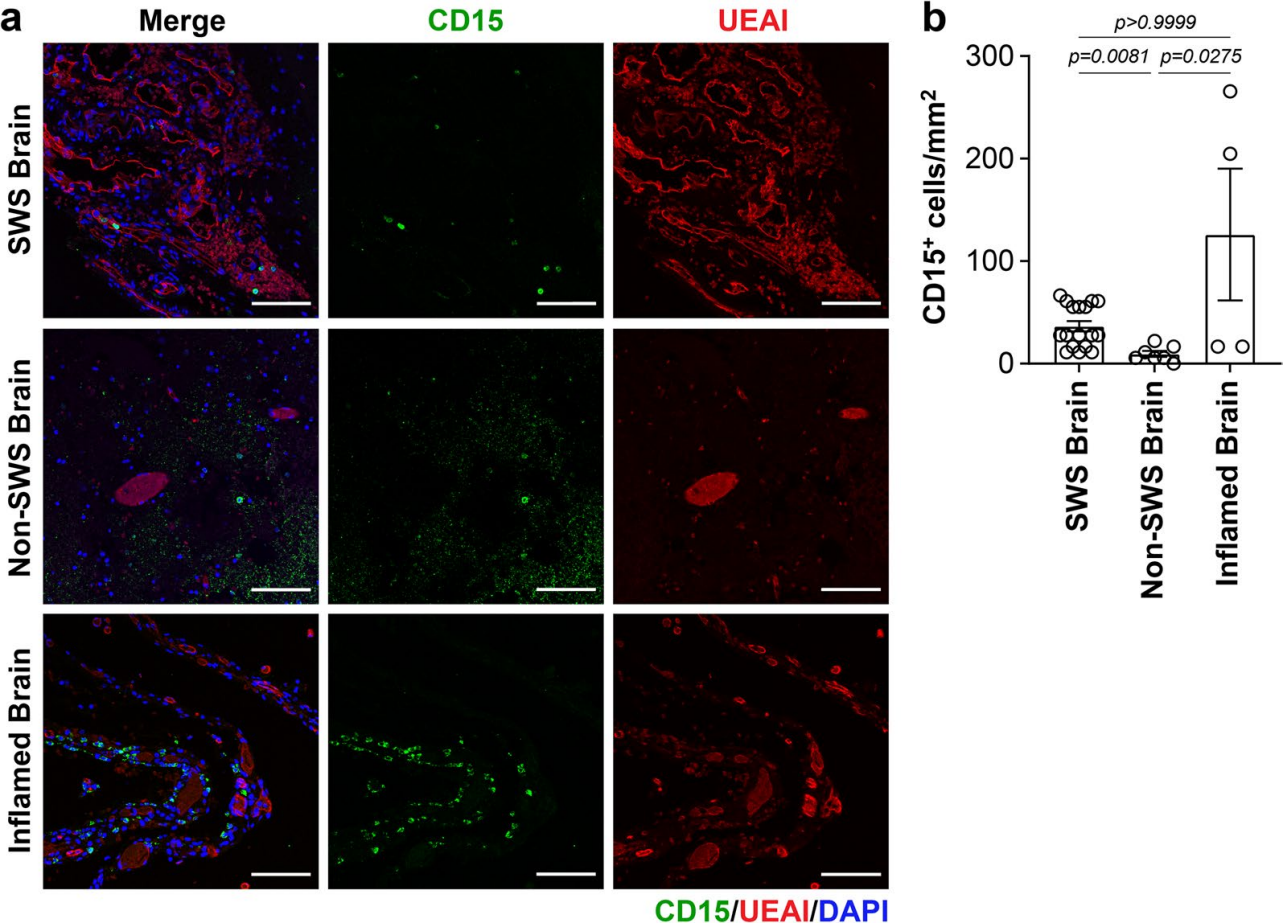
CD15^+^ neutrophils in the Sturge-Weber brain. **a** CD15 (green), UEAI (red) and counterstaining for DAPI (blue) in SWS brain (top), non-SWS brain (middle), and inflamed brain (bottom). Scale bar = 50 µm. **b** Quantification of CD15^+^ cells (right) in the SWS brain (n = 4), non-SWS brain (n = 2) and inflamed brain (n = 1) specimens. The p-values were calculated by One-way ANOVA test with Dunn’s multiple comparison test

To get a better grasp of the inflammatory cells present in CM-affected vascular beds, we stained for neutrophils using anti-CD15 antibody. CD15^+^ neutrophils were detected in CM-affected tissue sections (Fig. 5a); however they were sparse compared to MRC1^+^ macrophages. While sparse, there was a significant increase in the number of neutrophils in SWS brain compared to non-SWS brain vascular beds (Fig. 5b), suggesting they may also be recruited to CM-affected vascular beds.

### EC-R183Q promotes myeloid cell adhesion under static and laminar flow conditions

**Fig. 6:**
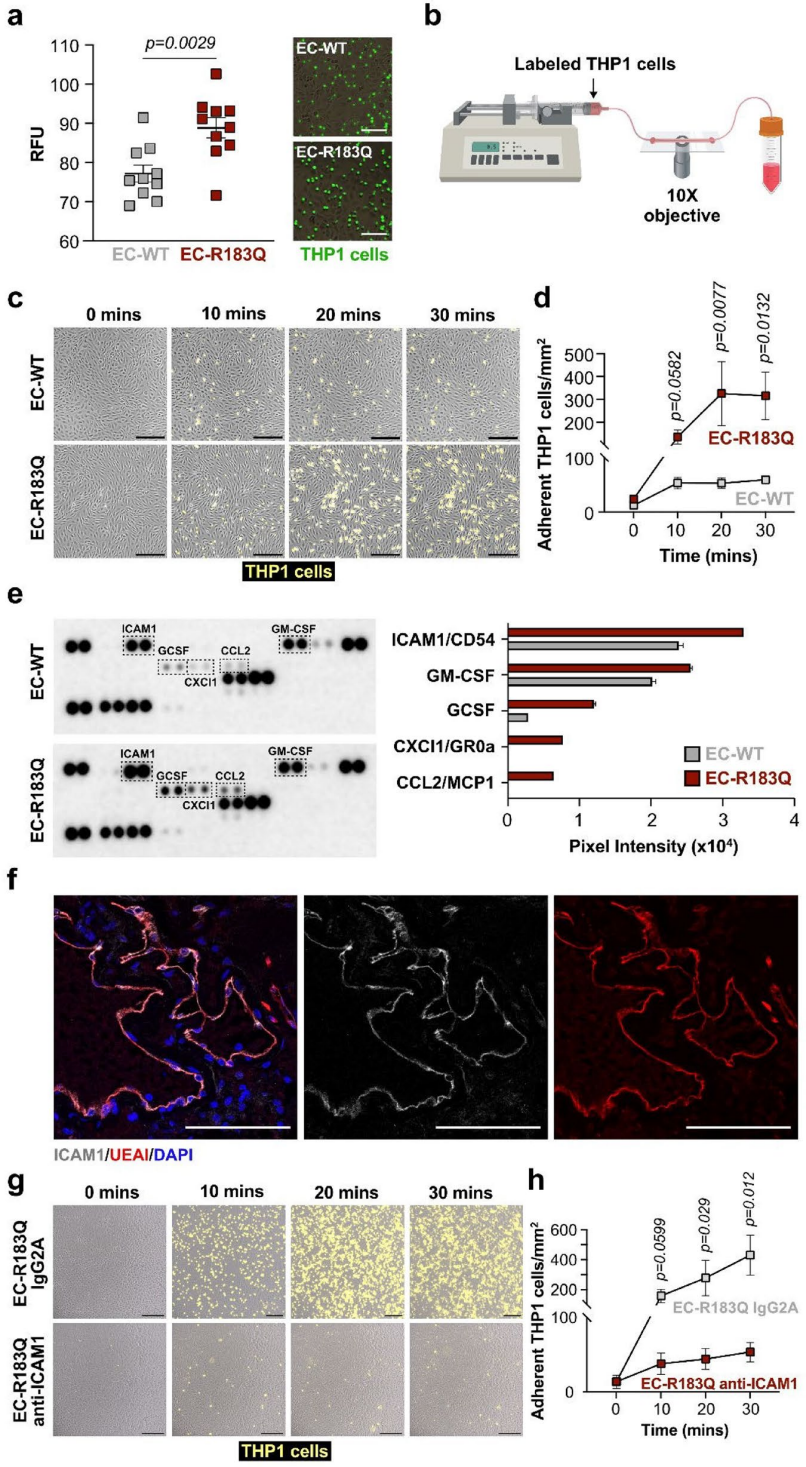
EC-R183Q promote significant THP1 cell adhesion under static and laminar flow-induced condition. **a** Fluorescence-labeled THP1 cells were incubated with EC-WT and EC-R183Q under static conditions (N = 10). Adherent cells were quantified after 1 h. *P*-value was calculated by two-tailed *t*-test. Phase-contrast images of EC-WT (top) or EC-R183Q (bottom) incubated with THP1 cells (green) at 1 h incubation. Scale bar = 50 µm. **b** Schematic of live-cell imaging set up. A flow rate of 0.5 ml/min was setup using a tabletop syringe pump with a 20 ml syringe Luer-Lock tip. After 5 min of recording, a switch system was used to deliver pre-stained THP1 cells under continuous uninterrupted flow for 30 min. **c** Time-lapse imaging of THP1 cells (yellow) adhesion to EC-WT (top), and EC-R183Q (bottom) under laminar flow. Images are at time point = 0, 10, 20, and 30 min. Scale bar = 200 µm. N = 6 independent experiments were performed. **d** Quantification of THP1 cell adhesion under flow over 10, 20, and 30 min. Mann Whitney test was performed to calculate p-value at each time point. **e** Proteome profiler cytokine array on conditioned media from EC-WT (top) and EC-R183Q (bottom) after incubation in 2% fetal bovine serum EBM2 media for 24 h. Altered protein levels between EC-WT and EC-R183Q are boxed. Protein levels were quantified by measuring dot intensity using FIJI (right). Three independent experiments were performed. **f** Intercellular adhesion molecule 1 (ICAM1, grey), UEAI (red), and nuclei counterstaining for DAPI (blue) in the SWS brain sections (n = 4). Scale bar = 50 µm. **g** Time-lapse imaging of THP1 cell (yellow) adhesion to EC-R183Q treated with IgG2A isotype control (top), and EC-R183Q treated with anti-ICAM1 antibody (bottom) under laminar flow. Images are at time point = 0, 10, 20, and 30 min. Scale bar = 200 µm. N = 5 independent experiments were performed. **h** Quantification of single cell tracking of THP1 cells under flow over 10, 20, and 30 min. Mann Whitney test was performed to calculate p-value at each time point

To investigate the mechanism by which macrophages move into the perivascular environment in the SWS brain, we used THP1 cells, a monocytic/macrophage cell line, to investigate the macrophage adhesion in vitro. Inflammation is characterized by a local increase of leukocyte recruitment into the inflamed area; the cells leave the circulation by rolling on, adhering to, and transmigrating across the endothelium. In our study, within one hour of incubation with THP1 cells, EC-R183Q had a significantly increased number of adherent THP1 cells compared to EC-WT (Fig. 6a). The increased adhesion to EC-R183Q was confirmed with a second monocytic/macrophage cell line called U937 (Additional file 5: Fig. S5). We next analyzed the interaction of THP1 cells with the endothelial monolayer under laminar flow conditions using live cell imaging (Fig. 6b). We observed significantly increased adherence of THP1 cells to the EC-R183Q monolayer compared to EC-WT over 30 min of laminar flow (Fig. 6c, Additional file 6: Fig. S6a, b). Single-cell tracking quantification revealed that THP1 cells show a three fold and six fold increase in adherence to EC-R183Q after 10 min and 20 min of flow (0.5 ml/min), respectively. The increased adherence remained elevated at 30 min (Fig. 6d).

To determine which cytokines and adhesion molecules might play a role in the increased adhesion, we performed a cytokine array on conditioned media from EC-R183Q and EC-WT cultured in reduce FBS (2%) containing media for 24 h (Fig. 6e). We found several proinflammatory cytokines (CCL2, CXCl1, GCSF, GM-CSF) and ICAM1/CD54 increased in EC-R183Q compared to EC-WT (Fig. 6e). To assess whether ICAM1 is present in ECs in CM vascular beds, we immune-stained CM in SWS brain sections for ICAM1. Indeed, ICAM1 was expressed on the endothelium of some, but not all, blood vessels in brain CMs specimens (N = 4), suggesting local variability of this adhesion molecule in the brain CM. (Fig. 6f). Next, we sought to test if we can prevent the increased THP1 cell adhesion by treating the EC-R183Q with anti-ICAM1 antibody prior to perfusing with labeled THP1 cells. We observed a significant decrease in THP1 cell adhesion upon pre-treatment with anti-ICAM1 compared to the IgG2A isotype control antibody (Fig. 6g, Additional file 6: Fig. S6c, d). Quantification of single cell tracking showed that treating EC-R183Q with anti-ICAM1 antibody decreased the THP1 cell adhesion to three fold and five fold after 10 min and 20 min, respectively, compared to IgG2A isotype antibody treated EC-R183Q (Fig. 6h). This strongly implicates ICAM1 as a mediator of the increased THP1 adhesion to the mutant EC-R183Q.

## Discussion

**Fig. 7:**
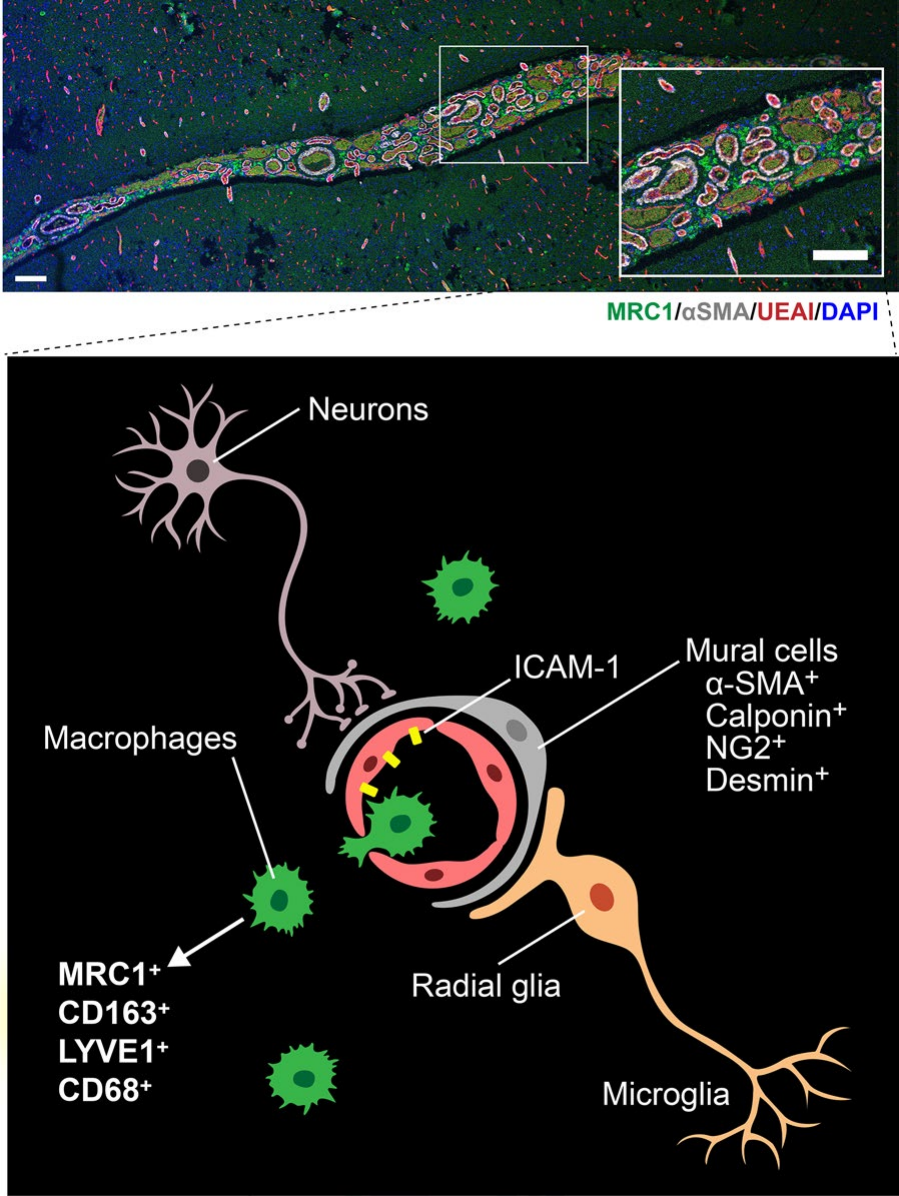
Sturge-Weber brain microenvironment. Tiling image (top) of Sturge-Weber brain leptomeninges labeled for MRC1 (green), αSMA (grey), UEAI (red) and nuclei counterstaining for DAPI (blue). Zoom insert shows vessels with and without αSMA layer surrounded by MRC1^+^ cells in the perivascular space. Scale bar = 200 µm. The illustration (bottom) shows mural cells (αSMA, calponin, NG2 and desmin) around the endothelial cells. MRC1, CD163, LYVE1, and CD68 macrophages in the perivascular space along with the neurons and microglia cells

Here we show CM in brain specimens from patients with SWS are populated with macrophage-like cells expressing MRC1, CD163, CD68, and LYVE1 (Fig. 7). We further show that macrophages adhere rapidly and robustly, under static and laminar flow conditions, to ECs expressing the *GNAQ* R183Q mutation. This suggests the mutant ECs facilitate transmigration of the MRC1^+^/CD163^+^/CD68^+^/LYVE1^+^ cells across the CM endothelium. We provide further information on characteristics of the CM vessels: they are enlarged, as expected, but unexpectedly, these enlarged vessels tend to lack mural cell coverage, compared to vessels in non-SWS brain. Other established features of CM vessels are active remodeling, permeability, and stasis. These phenotypic characteristics may provide guideposts for understanding the pathology and downstream consequences of CM in the brain.

Macrophages are versatile immune cells and are crucial for homeostasis. They can sense and respond to pathogens and participate in tissue repair after injury. They are known to play diverse roles in development, acute response to injury, and tissue repair. MRC1/CD206, a receptor expressed by tissue resident macrophages, is required to mediate endocytosis of glycoproteins. These mannose receptors recognize complex sugars on the surface of pathogens and facilitate phagocytosis. Interestingly, our findings demonstrate abundant MRC1^+^ cells in perivascular space of SWS brain, which could potentially be scavenging and engulfing cells and cellular debri. LYVE1 is known to be expressed by lymphatic endothelium however recent studies also show non-endothelial cells express LYVE1 in certain tissues. It has been previously reported that LYVE1 positive cells are expressed by a subset of macrophages [3, 19, 30]. One study reported that LYVE1 and CD68 positive cells are present in rat meninges and these unique subsets of cells are present in both lymphatic and non-lymphatic regions [3]. In this study, we found > 90% of MRC1^+^ co-express LYVE1 and CD68. Recent studies in zebrafish shed light on an interesting perivascular cell population called by several different names including perivascular macrophages, mural lymphatic ECs, fluorescent granular perithelial cells, or Mato cells; all of which express MRC1 and LYVE1 [27].

An important question is where these perivascular macrophages originate from. The slow blood flow in CM may contribute to higher physical retention of leukocytes in general. One possibility is that these macrophages may be recruited from the bloodstream and extravasate at CM-affected vessels via adhesion molecules such as ICAM1 which we find expressed on brain CM vessels. Recent studies have shown that mechanical forces may play a role via PIEZO1 and G_q_/G_ll_, where slow flow and ICAM1 together activate the PIEZO1 channel [1]. In slow flow CM, ICAM1 may be critical in inducing and initiating macrophage diapedeses [29]. Growing evidence has shown that endothelial ICAM1 hotspots function to limit vascular leakage during inflammation induced leukocyte extravasation and furthermore, a reduction in ICAM1 specific hotspots increases vascular leakage [13, 26]. It is also possible that mechanisms similar to the recruitment of pro-angiogenic monocytes into tumors are at play. A recent study showed that inflammatory and angiogenic factors together, but not alone, lead to the specific recruitment of alternatively activated monocytes into solid tumors [23]. Vessel leakiness may also play a role in the macrophage transmigration into the perivascular space where mutant ECs may fail to form tight junctions. Future studies using cellular models of CMs should address the interactions with and possible recruitment of monocyte populations to CM-affected blood vessels. Another possibility for future investigation is whether macrophages could differentiate and proliferate from locally present monocytes in the CM microenvironment.

More studies are needed to determine the role of alternatively activated macrophages in CM vascular beds. Given their marker profile and the inflammatory microenvironment, it is possible that they contribute to perpetuating angiogenesis and vascular remodeling. Indeed, the marker profile suggests that these cells could promote angiogenesis, as observed in different contexts. For example, LYVE1^+^ macrophages are crucial for dense vascular network formation in adult mouse adipose tissue [4], and the number of CD163^+^ macrophages in tumors is associated with increased angiogenesis [5]. Alternatively activated macrophages expressing MRC1, TIE2 and CXCR4 also stimulate tumor relapse in a model with Lewis lung carcinoma cells and promote angiogenesis via VEGFA secretion [16]. In CMs, we now know that *GNAQ* R183Q mutant ECs produce high levels of ANGPT2 [12, 14], an angiogenic factor that may participate in promoting alternatively proangiogenic macrophage accumulation. ECs can secrete factors that directly influence macrophage polarization and localization. For example, endothelial-derived IL6 can induce alternative macrophage polarization in glioblastoma [28]. ECs can also selectively allow transmigration of proangiogenic monocytes in the presence of pro-angiogenic (VEGFA, ANGPT2) and pro-inflammatory (ICAM1, TNFα, IL1β) factors [23].

Here we have established that blood vessels in CM-affected vascular beds are leaky, activated, and contain a higher number of MRC1^+^ macrophages and CD15^+^ neutrophils than non- CM brain vascular beds. We also know from previous studies [9, 15, 24] that the *GNAQ* R183Q mutation is enriched in ECs. The question remains as to the extent and location of mutant ECs along vessels lumens and further, the extent and identity of non- mutant ECs that maybe in the vicinity. An interesting open question is whether *GNAQ* R183Q mutant ECs influence the polarization of locally present macrophages and monocytes. Another question that remains is whether the mutant ECs are found in clusters, or are they dispersed throughout the CM-affected blood vessels. At present there are no reliable markers or tools to distinguish in situ R183Q mutant ECs from non-mutant EC. The identity and location of non-mutant ECs will provide clues about disease origin, ontogeny, and progression.

There are several limitations to this study. First, we used a small number of SWS brain specimens and non-SWS controls. Despite the limited number of specimens this study provides a valuable snapshot of CM in progress. The human samples analyzed here are highly relevant as they provide an insight into the structure of SWS-affected vascular beds. MRC1^+^ cells were found in the vicinity of both mural cell covered and uncovered vessels, which we did not specifically quantify. Thus, it remains unclear if there is a preferential localization of the macrophage-like cells. Future studies using CM specific animal models may address this in a specific manner. A second limitation is the in vitro studies, CRISPR editing of one *GNAQ* allele in telomerase immortalized human aortic ECs was used and clones were selected to yield cell populations that were 100% mutant. Thus, there was a lack of mosaicism in the in vitro setup, which is in contrast to the human disease. SWS brain isolated ECs under culture and expansion shows mutant allelic frequency to be between 15 and 21%, which indicates a mixture of mutant and non-mutant ECs [15]. Regardless of using telomerase modified ECs, our findings expand our understanding of CM and could potentially provide a starting point for drug testing and other functional studies. A final limitation is that THP1 and U937 cells may not recapitulate the unique phenotype of macrophages expressing MRC1^+^/CD68^+^/LYVE1^+^. Regardless, they provide first insights into how mutant ECs interact with a monocyte/macrophage cell line. In vivo and in vitro studies to study the mechanism of macrophage recruitment should be further explored.

In conclusion, the role of MRC1^+^/CD68^+^/LYVE1^+^ macrophages should be further studied in the setting of CMs that occur in patients with SWS. It is possible that the macrophages described here contribute to the disease progression or they may mitigate adverse impacts of the CM by clearing cells and cellular debri.

## Supplementary Information

**Additional file 1: Fig. S1.** MRC1+ cells quantified in individual brain specimens. Top x-axis shows % mutant allelic frequency (MAF) of the corresponding samples measured by droplet digital PCR. ‘–’ indicates the mutant allele was not detected.

**Additional file 2: Fig. S2.** MRC1+ cells in SWS brain express CD163. CD163 (yellow), MRC1 (grey), UEAI (red), and counterstaining for DAPI (blue) in SWS brain specimens. Separate channels of CD163 (top left), MRC1 (top right), UEAI (bottom left) and DAPI (bottom right). SWS brain specimens (n = 4). Scale bar = 50 µm.

**Additional file 3: Fig. S3.** Phagocytic MRC1+ cells observed in SWS brain sections. MRC1 (green), autofluorescence red blood cells (RBCs, red), UEAI (grey), and nuclei counterstaining for DAPI (blue). White arrows point to RBCs and MRC1+ cells in close proximity in the perivascular space. SWS brain specimens (n = 4). Scale bar = 50 µm.

**Additional file 4: Fig. S4.** MRC1+ cells are distinct from α-SMA+ cells in the Sturge-Weber brain. αSMA (grey), MRC1 (green), UEAI (red), and counterstaining for DAPI (blue). Separate channels of αSMA (top left), MRC1 (top right), UEAI (bottom left) and DAPI (bottom right). SWS brain specimens (n = 4). Scale bar = 50 µm.

**Additional file 5: Fig. S5.** Adhesion of the monocytic cell line U937 to EC-WT and EC-R183Q. Fluorescence-labeled U937 cells were incubated with EC-WT and EC-R183Q under static conditions (N=10). Adherent cells were quantified after 1 h. The *p*-value was calculated by two-tailed *t*-test.

**Additional file 6: Fig. S6.** Tile images at the end of 30 minutes of live cell imaging. **a** EC-WT **b** EC-R183Q **c** EC-R183Q+ IgG2A isotype control **d** EC-R183Q+anti-ICAM1. Scale bar = 200 µm.

**Additional file 7: Table S1.** List of antibodies and other reagents.

**Additional file 8: Table S2.** List of ddPCR primers and probes.
