# Correction: MRC1 and LYVE1 expressing macrophages in vascular beds of GNAQ p.R183Q driven capillary malformations in Sturge Weber syndrome

**DOI:** 10.1186/s40478-026-02326-7

**Published:** 2026-06-12

**Authors:** Sana Nasim, Colette Bichsel, Stephen Dayneka, Robert Mannix, Annegret Holm, Mathew Vivero, Sanda Alexandrescu, Anna Pinto, Arin K. Greene, Donald E. Ingber, Joyce Bischoff

**Affiliations:** 1 Vascular Biology Program, Boston Children’s Hospital and Harvard Medical School, Boston, MA 02115 USA; 2 Department of Surgery, Boston Children’s Hospital and Harvard Medical School, Boston, MA 02115 USA; 3 CSEM SA, Hegenheimermattweg 167 A, 4123 Allschwil, Switzerland; 4 Department of Plastic and Oral Surgery, Boston Children’s Hospital and Harvard Medical School, Boston, MA 02115 USA; 5 Department of Pathology, Boston Children’s Hospital and Harvard Medical School, Boston, MA 02115 USA; 6 Department of Neurology, Boston Children’s Hospital and Harvard Medical School, Boston, MA 02115 USA; 7 Wyss Institute for Biologically Inspired Engineering at Harvard University, Boston, MA 02215 USA; 8 Harvard John A. Paulson School of Engineering and Applied Sciences, Harvard University, Cambridge, MA 02139 USA

**Correction to: Acta Neuropathologica Communications (2024) 12:47** 10.1186/s40478-024-01757-4

In this article [1], Fig. 6 appeared incorrectly and have now been corrected in the original publication. For completeness and transparency, both correct and incorrect versions are displayed below.

Incorrect Fig. 6

**Fig. 6:**
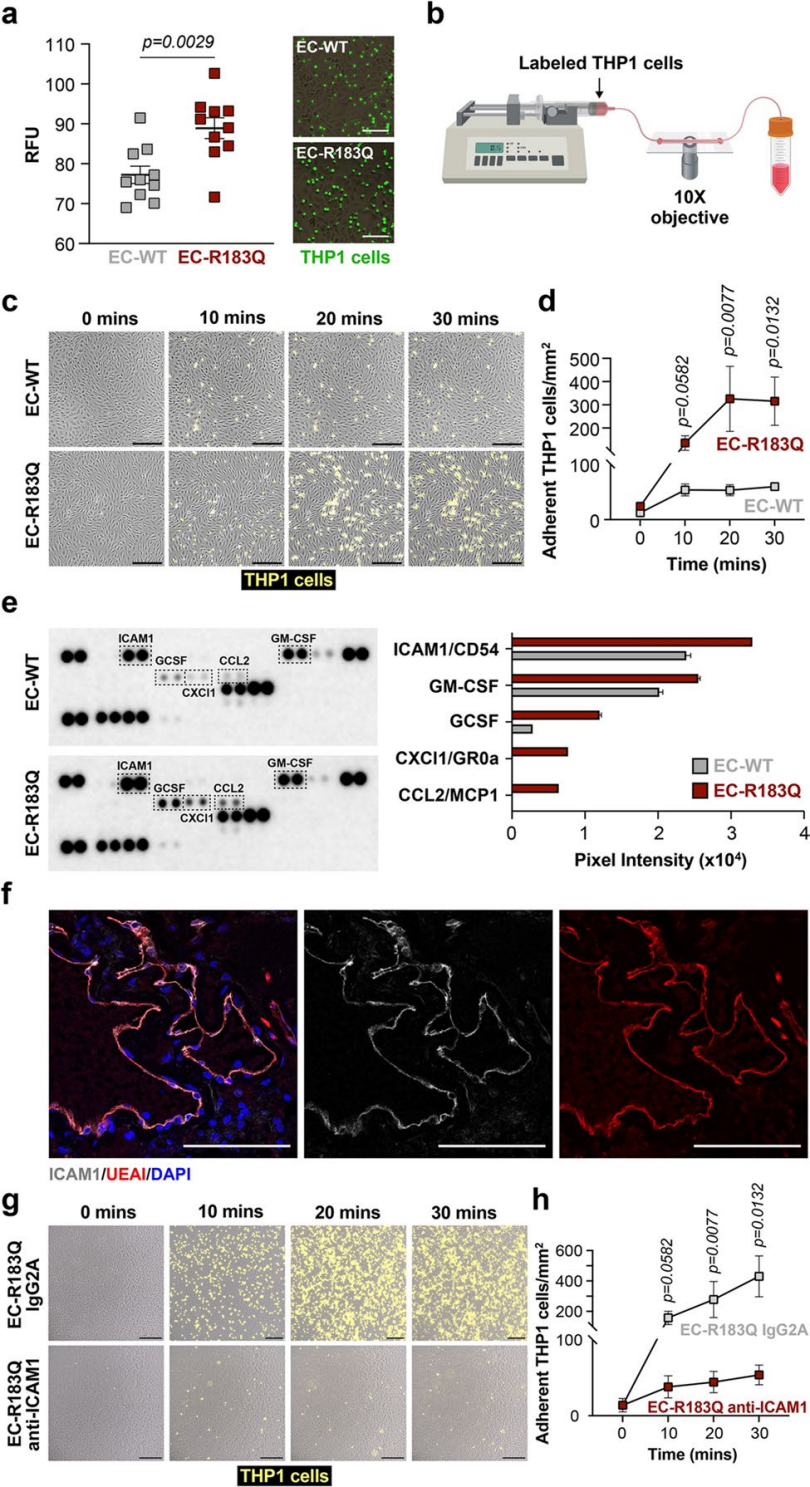
EC-R183Q promote significant THP1 cell adhesion under static and laminar flow-induced condition. **a** Fluorescence-labeled THP1 cells were incubated with EC-WT and EC-R183Q under static conditions (N = 10). Adherent cells were quantified after 1 h. *P*-value was calculated by two-tailed t-test. Phase-contrast images of EC-WT (top) or EC-R183Q (bottom) incubated with THP1 cells (green) at 1 h incubation. Scale bar = 50 μm. **b** Schematic of live-cell imaging set up. A flow rate of 0.5 ml/min was setup using a tabletop syringe pump with a 20 ml syringe Luer-Lock tip. After 5 min of recording, a switch system was used to deliver pre-stained THP1 cells under continuous uninterrupted flow for 30 min. **c** Time-lapse imaging of THP1 cells (yellow) adhesion to EC-WT (top), and EC-R183Q (bottom) under laminar flow. Images are at time point = 0, 10, 20, and 30 min. Scale bar = 200 μm. N = 6 independent experiments were performed. **d** Quantification of THP1 cell adhesion under flow over 10, 20, and 30 min. Mann Whitney test was performed to calculate *p*-value at each time point. **e** Proteome profiler cytokine array on conditioned media from EC-WT (top) and EC-R183Q (bottom) after incubation in 2% fetal bovine serum EBM2 media for 24 h. Altered protein levels between EC-WT and EC-R183Q are boxed. Protein levels were quantified by measuring dot intensity using FIJI (right). Three independent experiments were performed. **f** Intercellular adhesion molecule 1 (ICAM1, grey), UEAI (red), and nuclei counterstaining for DAPI (blue) in the SWS brain sections (n = 4). Scale bar = 50 μm. **g** Time-lapse imaging of THP1 cell (yellow) adhesion to EC-R183Q treated with IgG2A isotype control (top), and EC-R183Q treated with anti-ICAM1 antibody (bottom) under laminar flow. Images are at time point = 0, 10, 20, and 30 min. Scale bar = 200 μm. N = 5 independent experiments were performed. **h** Quantification of single cell tracking of THP1 cells under flow over 10, 20, and 30 min. Mann Whitney test was performed to calculate *p*-value at each time point

Correct Fig. 6

**Fig. 6:**
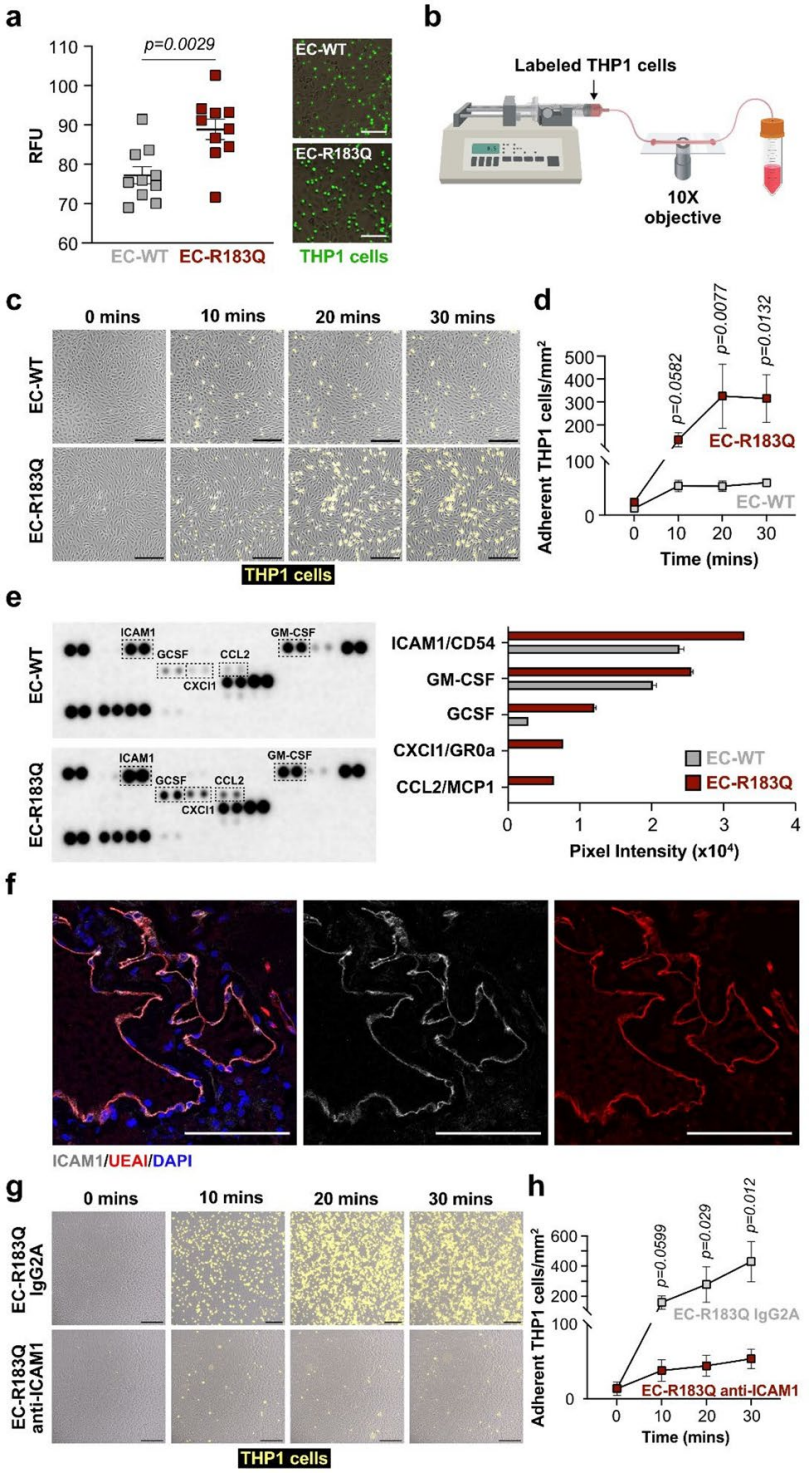
EC-R183Q promote significant THP1 cell adhesion under static and laminar flow-induced condition. **a** Fluorescence-labeled THP1 cells were incubated with EC-WT and EC-R183Q under static conditions (N = 10). Adherent cells were quantified after 1 h. *P*-value was calculated by two-tailed t-test. Phase-contrast images of EC-WT (top) or EC-R183Q (bottom) incubated with THP1 cells (green) at 1 h incubation. Scale bar = 50 μm. **b** Schematic of live-cell imaging set up. A flow rate of 0.5 ml/min was setup using a tabletop syringe pump with a 20 ml syringe Luer-Lock tip. After 5 min of recording, a switch system was used to deliver pre-stained THP1 cells under continuous uninterrupted flow for 30 min. **c** Time-lapse imaging of THP1 cells (yellow) adhesion to EC-WT (top), and EC-R183Q (bottom) under laminar flow. Images are at time point = 0, 10, 20, and 30 min. Scale bar = 200 μm. N = 6 independent experiments were performed. **d** Quantification of THP1 cell adhesion under flow over 10, 20, and 30 min. Mann Whitney test was performed to calculate *p*-value at each time point. **e** Proteome profiler cytokine array on conditioned media from EC-WT (top) and EC-R183Q (bottom) after incubation in 2% fetal bovine serum EBM2 media for 24 h. Altered protein levels between EC-WT and EC-R183Q are boxed. Protein levels were quantified by measuring dot intensity using FIJI (right). Three independent experiments were performed. **f** Intercellular adhesion molecule 1 (ICAM1, grey), UEAI (red), and nuclei counterstaining for DAPI (blue) in the SWS brain sections (n = 4). Scale bar = 50 μm. **g** Time-lapse imaging of THP1 cell (yellow) adhesion to EC-R183Q treated with IgG2A isotype control (top), and EC-R183Q treated with anti-ICAM1 antibody (bottom) under laminar flow. Images are at time point = 0, 10, 20, and 30 min. Scale bar = 200 μm. N = 5 independent experiments were performed. **h** Quantification of single cell tracking of THP1 cells under flow over 10, 20, and 30 min. Mann Whitney test was performed to calculate *p*-value at each time point

The original article has been corrected.
