# Supplementary figures and images for: MRC1 and LYVE1 expressing macrophages in vascular beds of GNAQ p.R183Q driven capillary malformations in Sturge Weber syndrome
Source: Acta Neuropathol Commun. 2024 Mar 26;12:47. doi: 10.1186/s40478-024-01757-4 (PMC10964691; doi:10.1186/s40478-024-01757-4)

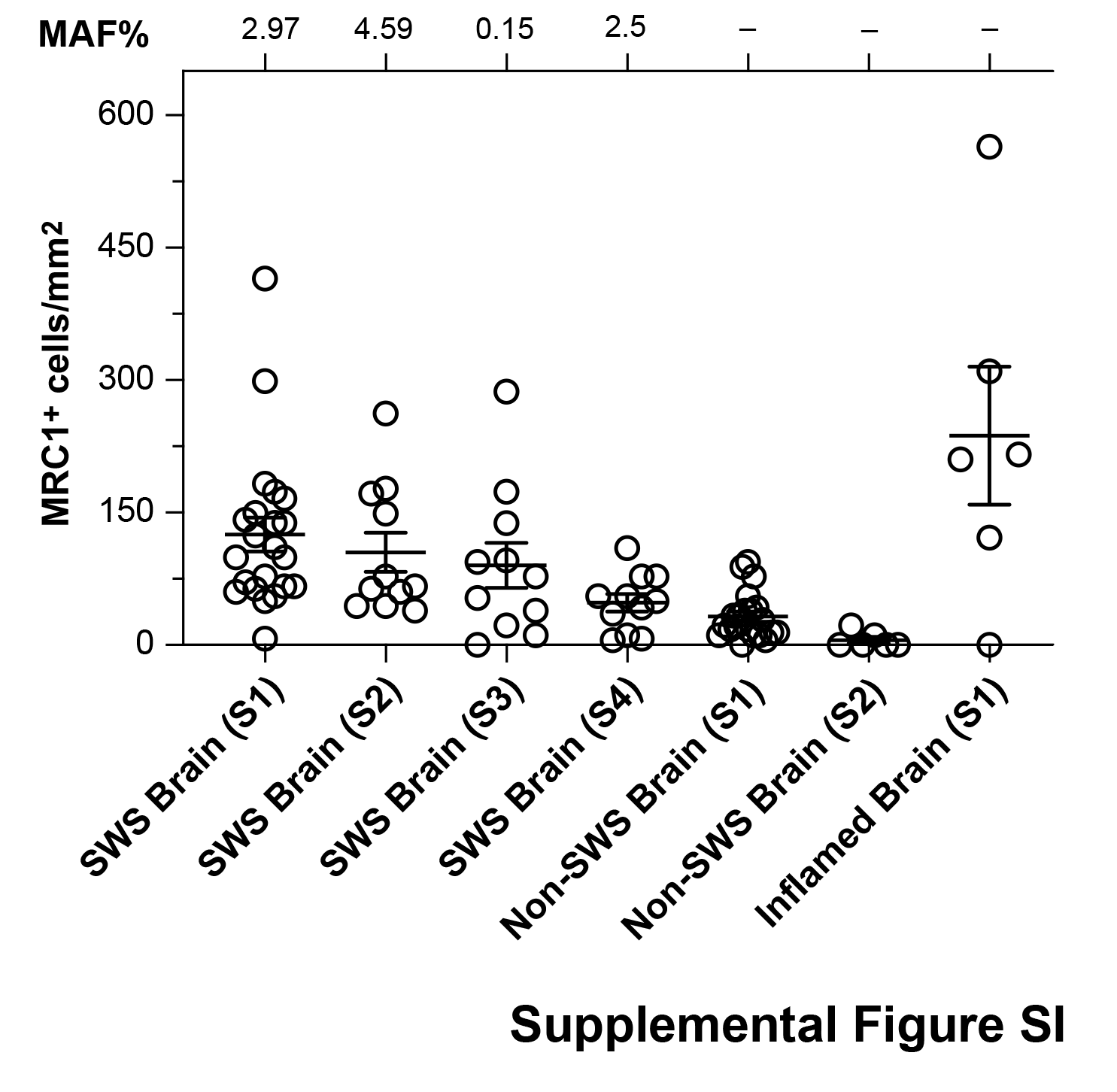

Supplement: Supplementary file 1 — Additional file 1: Fig. S1. MRC1+ cells quantified in individual brain specimens. Top x-axis shows % mutant allelic frequency (MAF) of the corresponding samples measured by droplet digital PCR. ‘–’ indicates the mutant allele was not detected. [file 40478_2024_1757_MOESM1_ESM.png]

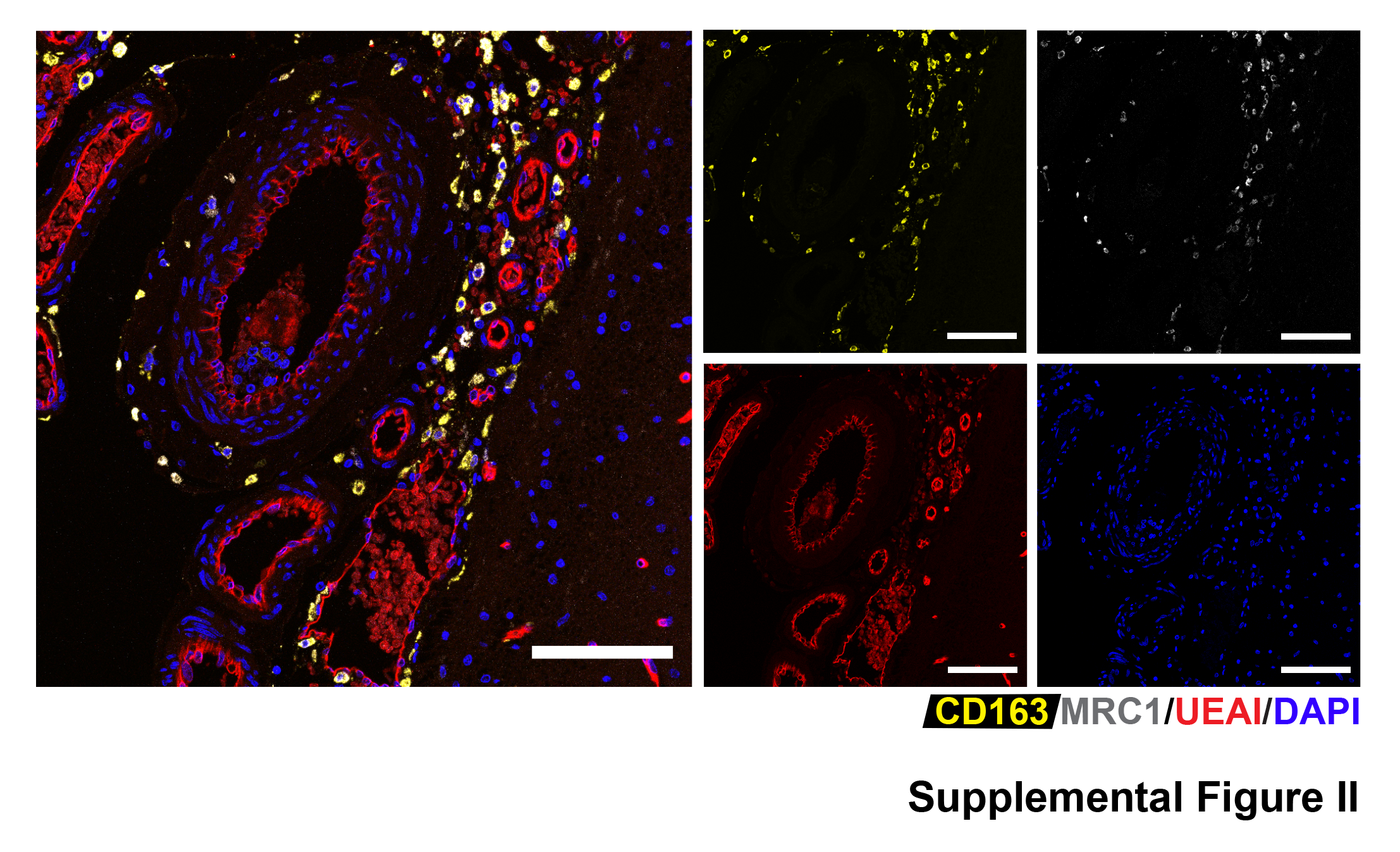

Supplement: Supplementary file 2 — Additional file 2: Fig. S2. MRC1+ cells in SWS brain express CD163. CD163 (yellow), MRC1 (grey), UEAI (red), and counterstaining for DAPI (blue) in SWS brain specimens. Separate channels of CD163 (top left), MRC1 (top right), UEAI (bottom left) and DAPI (bottom right). SWS brain specimens (n = 4). Scale bar = 50 µm. [file 40478_2024_1757_MOESM2_ESM.png]

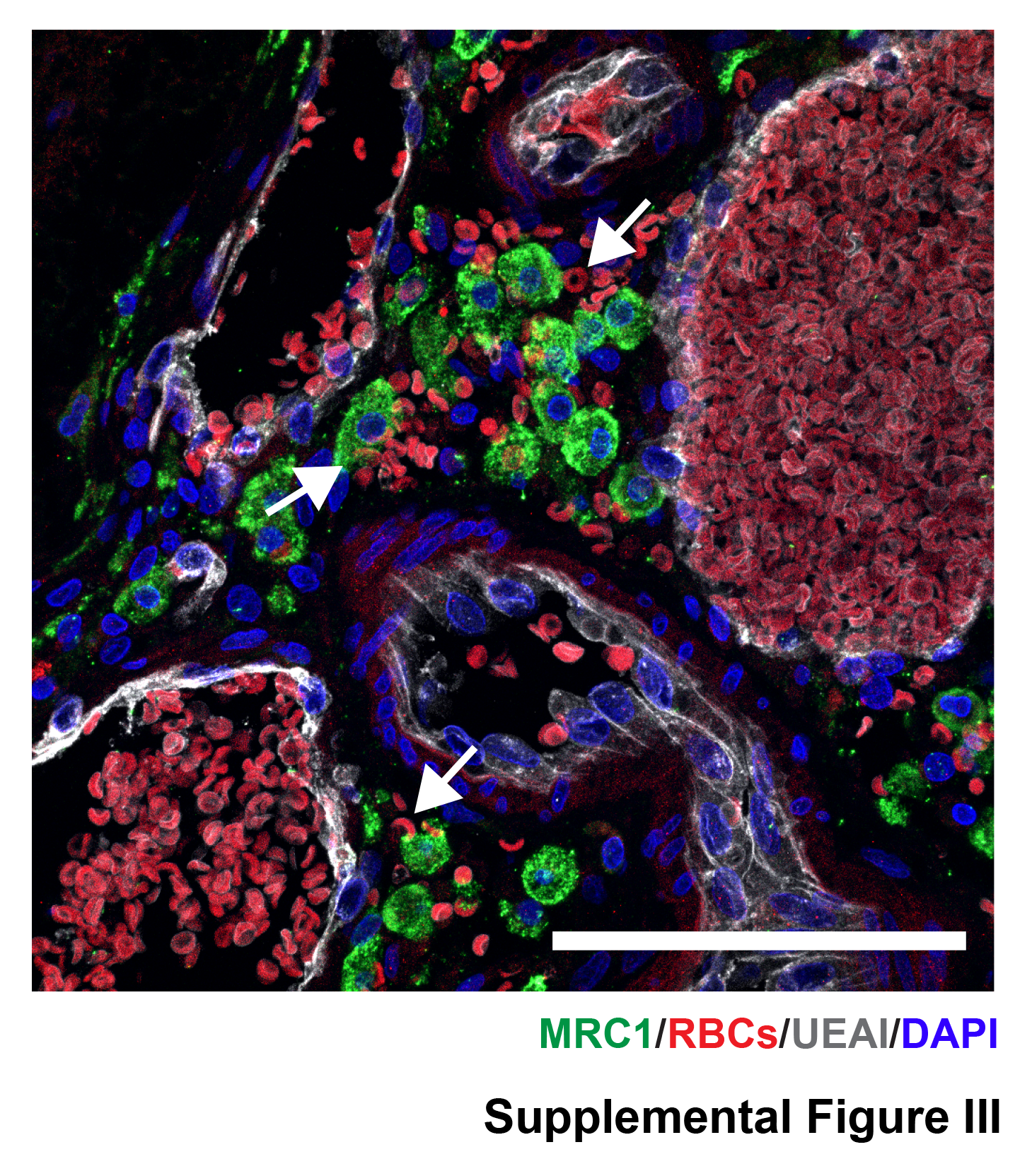

Supplement: Supplementary file 3 — Additional file 3: Fig. S3. Phagocytic MRC1+ cells observed in SWS brain sections. MRC1 (green), autofluorescence red blood cells (RBCs, red), UEAI (grey), and nuclei counterstaining for DAPI (blue). White arrows point to RBCs and MRC1+ cells in close proximity in the perivascular space. SWS brain specimens (n = 4). Scale bar = 50 µm. [file 40478_2024_1757_MOESM3_ESM.png]

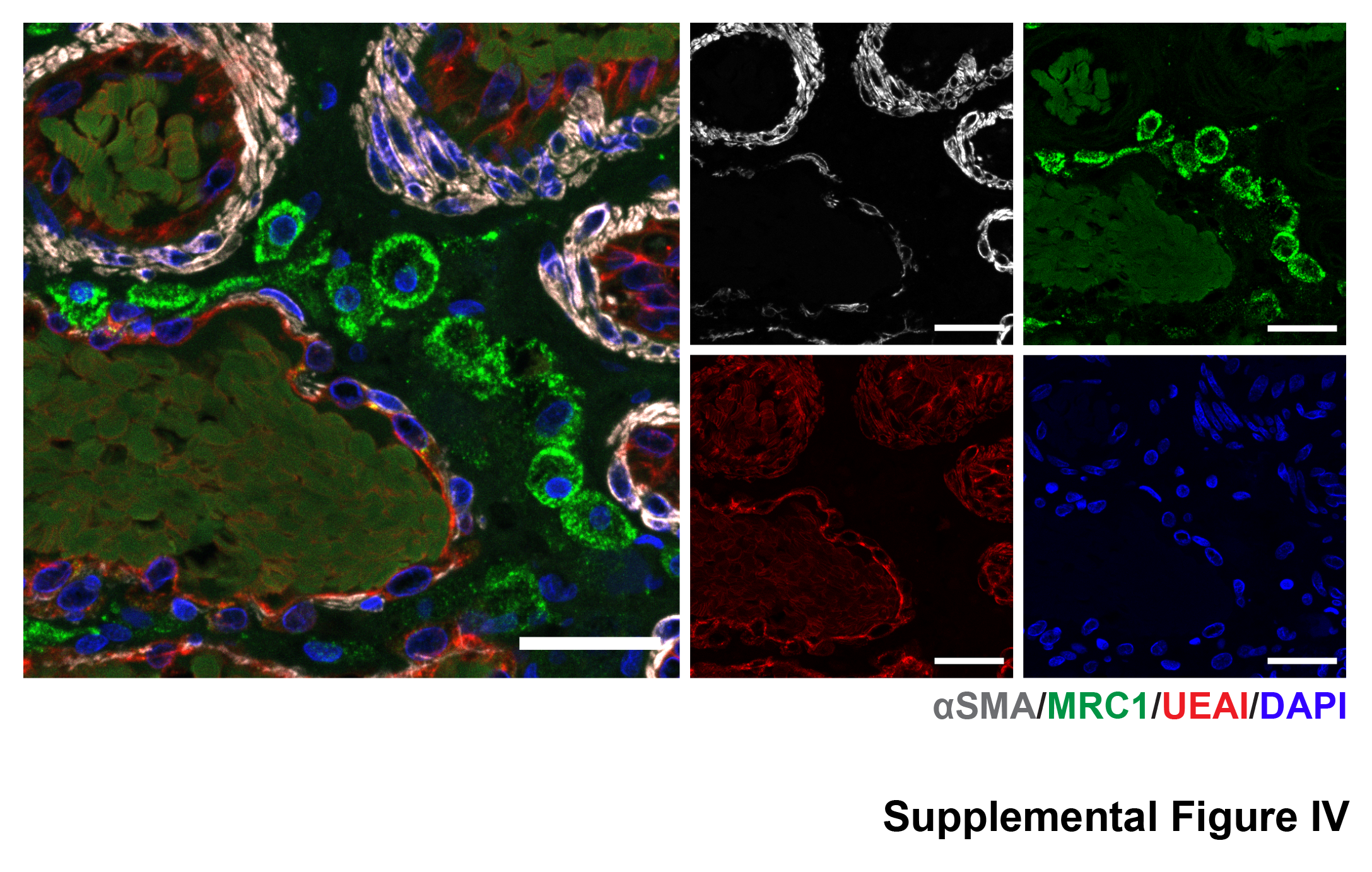

Supplement: Supplementary file 4 — Additional file 4: Fig. S4. MRC1+ cells are distinct from α-SMA+ cells in the Sturge-Weber brain. αSMA (grey), MRC1 (green), UEAI (red), and counterstaining for DAPI (blue). Separate channels of αSMA (top left), MRC1 (top right), UEAI (bottom left) and DAPI (bottom right). SWS brain specimens (n = 4). Scale bar = 50 µm. [file 40478_2024_1757_MOESM4_ESM.png]

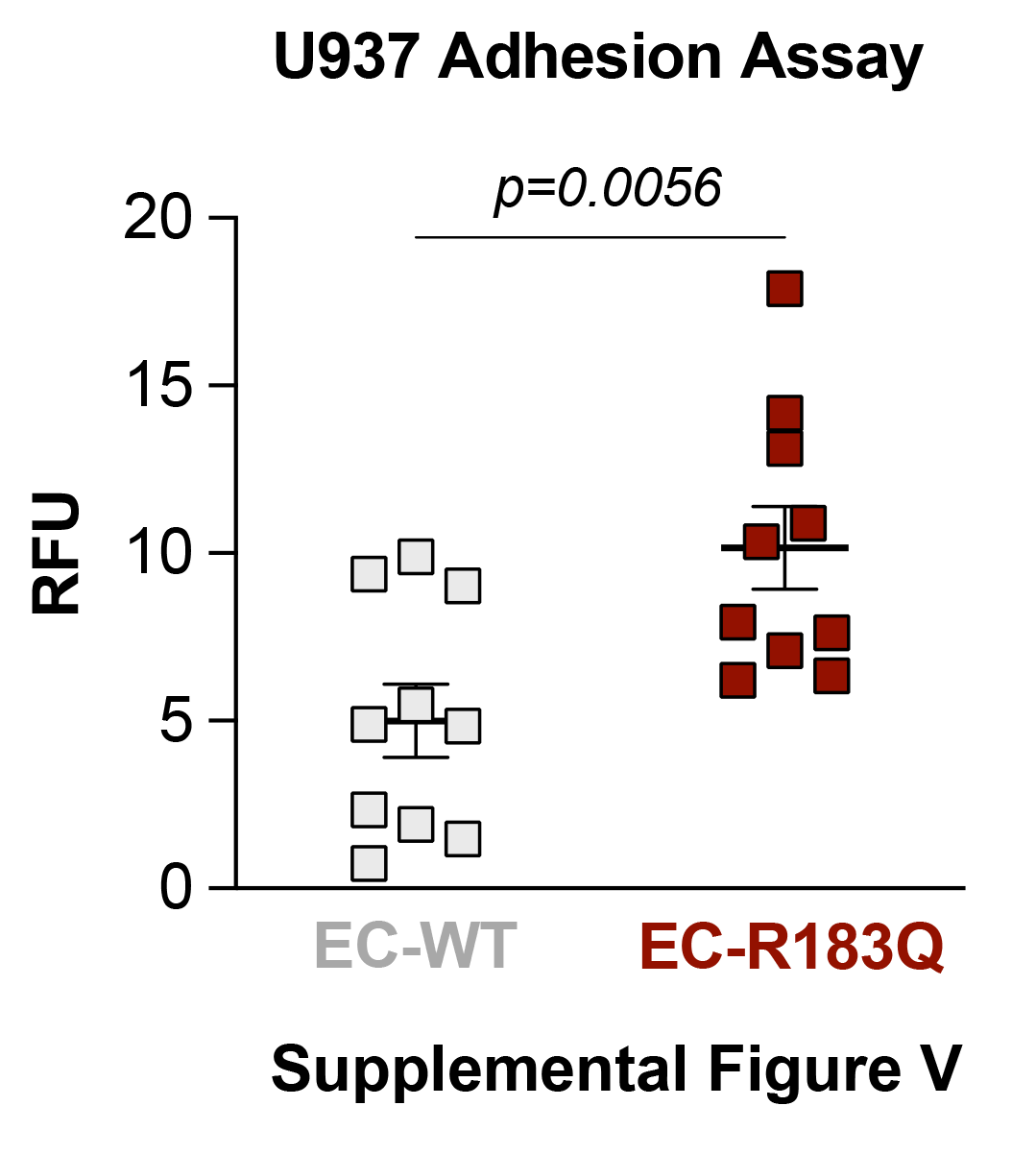

Supplement: Supplementary file 5 — Additional file 5: Fig. S5. Adhesion of the monocytic cell line U937 to EC-WT and EC-R183Q. Fluorescence-labeled U937 cells were incubated with EC-WT and EC-R183Q under static conditions (N=10). Adherent cells were quantified after 1 h. The p-value was calculated by two-tailed t-test. [file 40478_2024_1757_MOESM5_ESM.png]

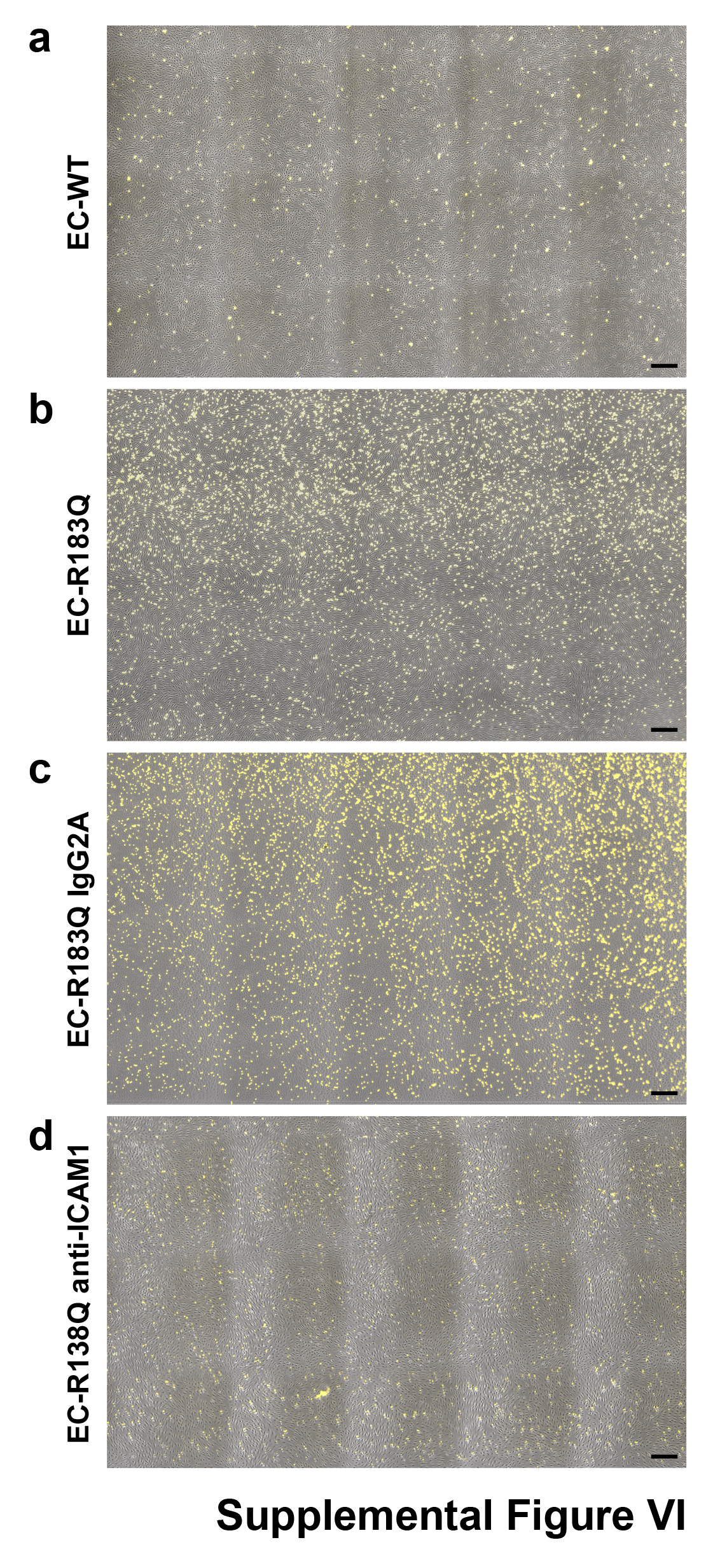

Supplement: Supplementary file 6 — Additional file 6: Fig. S6. Tile images at the end of 30 minutes of live cell imaging. a EC-WT b EC-R183Q c EC-R183Q+ IgG2A isotype control d EC-R183Q+anti-ICAM1. Scale bar = 200 µm. [file 40478_2024_1757_MOESM6_ESM.png]
