# Supplementary material for: MRC1 and LYVE1 expressing macrophages in vascular beds of GNAQ p.R183Q driven capillary malformations in Sturge Weber syndrome
Source: Acta Neuropathol Commun. 2024 Mar 26;12:47. doi: 10.1186/s40478-024-01757-4 (PMC10964691; doi:10.1186/s40478-024-01757-4)
Supplement: Supplementary file 7 — Additional file 7: Table S1. List of antibodies and other reagents. [file 40478_2024_1757_MOESM7_ESM.docx]

**Table S1:** *List of antibodies and other reagents*

| **Antibody** | **Source** | **Dilution** | **Vendor** | **Catalog Number** |
| --- | --- | --- | --- | --- |
| *Primary Antibodies* | | | | |
| Mannose receptor - MRC1 | Rabbit | 1:100 | abcam | ab64693 |
| Mannose receptor - MRC1 | Mouse | 1:100 | Millipore Sigma | AMAB90746 |
| NG2 | Rabbit | 1:100 | abcam | ab183929 |
| Desmin | Rabbit | 1:100 | abcam | ab15200 |
| ICAM1 (G-5) | Mouse | 1:50 | Santa Cruz | sc-8439 |
| Alpha smooth muscle actin – Cy3 conjugated | Mouse | 1:500 | Sigma Aldrich | C6198 |
| CD68 | Mouse | 1:100 | Invitrogen | 14-0688-82 |
| Ki67 | Rabbit | 1:100 | abcam | ab15580 |
| LYVE1 | Goat | 1:150 | R&D | AF2089SP |
| CD15 (HI98 clone) | Mouse | 1:100 | BioLegend | 301902 |
| CD163 (215927 clone) | Mouse | 1:50 | R&D | MAB1607 |
| Calponin | Rabbit | 1:100 | abcam | ab46794 |
| UEAI-Rhodamine | - | 1:100 | Vector labs | RL-1062 |
| *Secondary Antibodies* | | | | |
| Goat anti-Rabbit IgG (H+L), Alexa Fluor™ 488 | - | 1:200 | Invitrogen | A11008 |
| Donkey anti-Mouse IgG (H+L), Alexa Fluor™ 488 | - | 1:200 | Invitrogen | A21202 |
| Donkey anti-Goat IgG (H+L), Alexa Fluor^TM^ 594 | - | 1:200 | Invitrogen | A32758 |
| Donkey anti-Mouse IgG (H+L), Alexa Fluor™ 647 | - | 1:200 | Invitrogen | A31571 |
| Donkey anti-Rabbit IgG (H+L), Alexa Fluor™ 647 | - | 1:200 | Invitrogen | A31573 |
| *Other* | | | | |
| ProLong™ Diamond Antifade Mountant | - | 1:1000 | Invitrogen | P36965 |
| NucBlue™ Fixed Cell ReadyProbes™ Reagent | - | - | Invitrogen | R37606 |
| CellTrackerTM Red CMTPX dye | - | 1mM stock (1:1000) | Thermo Fisher Scientific | 34552 |
| VivoMAb anti-human CD54 (ICAM1)- (R-6.5-D6 clone) | - | 10ug/ml | BioXCell | BE0020-2 |
| InVivoMAb IgG2a isotype control - (C1.18.4 clone) | - | 10ug/ml | BioXCell | BE0085 |
