# Supplementary material for: MRC1 and LYVE1 expressing macrophages in vascular beds of GNAQ p.R183Q driven capillary malformations in Sturge Weber syndrome
Source: Acta Neuropathol Commun. 2024 Mar 26;12:47. doi: 10.1186/s40478-024-01757-4 (PMC10964691; doi:10.1186/s40478-024-01757-4)
Supplement: Supplementary file 8 — Additional file 8: Table S2. List of ddPCR primers and probes. [file 40478_2024_1757_MOESM8_ESM.docx]

**Table S2:** *List of ddPCR primers and probes*

| Primers/probes | Sequence |
| --- | --- |
| GNAQ R183Q forward primer | 5’-CCTGCCTACGCAACAAGAT-3’ |
| GNAQ R183Q reverse primer | 5’-GTAAGTCAAAGGGGTATTCGAT-3 |
| GNAQ R183Q reference primer | 5’-/5HEX/TGCTTAGAG/ZEN/TTCGAGTCCCCACC/3IABkFQ/-3’ |
| GNAQ R183Q mutant primer | 5’-/56-FAM/TGCTTAGAG/ZEN/TTCAAGTCCCCACC/3IABkFQ/-3’ |
